# Saturation Gap During Anesthesia in Glucose-6-Phosphate Dehydrogenase Deficiency and Methemoglobinemia: A Case Report and Review of Perioperative Management

**DOI:** 10.7759/cureus.107030

**Published:** 2026-04-14

**Authors:** Bruno Santiago, Gustavo M de Sousa, Vitor Felippe, Guilherme Q Bersot, Carlos Darcy A Bersot, Marcos A Lessa

**Affiliations:** 1 Anesthesia, Rio de Janeiro State University, Rio de Janeiro, BRA; 2 Anesthesia, Federal University of Rio de Janeiro, Rio de Janeiro, BRA; 3 Anesthesia, Brazilian National Cancer Institute, Rio de Janeiro, BRA; 4 Medicine, Faculty of Medicine of Campos, Campos dos Goytacazes, BRA; 5 Translational Medicine, Federal University of São Paulo, São Paulo, BRA; 6 Anesthesia, University of Iowa Hospitals and Clinics, Iowa City, USA

**Keywords:** anesthetic safety, congenital methemoglobinemia, co-oximetry, dyshemoglobinemia, glucose-6-phosphate dehydrogenase (g6pd) deficiency, propofol, remifentanil, saturation gap

## Abstract

The coexistence of glucose-6-phosphate dehydrogenase (G6PD) deficiency and methemoglobinemia may produce misleading pulse oximetry readings, creating a significant diagnostic challenge during the perioperative assessment of oxygenation. Methemoglobinemia results from the oxidation of hemoglobin iron to the ferric state, impairing oxygen delivery and altering light absorption, often leading to falsely low oxygen saturation values. A 20-year-old man with confirmed G6PD deficiency and suspected chronic methemoglobinemia underwent an elective electrophysiologic study for recurrent supraventricular tachycardia. On arrival in the operating room, pulse oximetry showed a saturation of 45% on room air, despite preserved clinical status. Arterial blood gas analysis revealed a partial pressure of oxygen (PaO₂) exceeding 400 mmHg, confirming adequate arterial oxygenation and establishing a saturation gap. General anesthesia was induced and maintained with propofol and remifentanil without complications. Due to the risk of hemolysis associated with methylene blue in G6PD deficiency, conservative management was adopted, with hyperbaric oxygen therapy available as a contingency strategy. The intraoperative course was uneventful, with stable hemodynamics and no evidence of tissue hypoxia or hemolysis. This case demonstrates that severe pulse oximetry desaturation does not necessarily reflect true hypoxemia. Recognition of a saturation gap is essential when oximetry findings are discordant with clinical presentation, particularly in patients with G6PD deficiency, in whom standard therapies may be contraindicated. Accurate evaluation requires the integration of clinical assessment with arterial blood gas analysis. This report highlights the diagnostic pitfalls and implications for safe perioperative management.

## Introduction

Unexpected pulse oximetry desaturation during anesthesia represents a critical diagnostic challenge, particularly when oxygenation parameters appear discordant with the patient's clinical condition. In such scenarios, dyshemoglobinemia, defined as the presence of abnormal hemoglobin species such as methemoglobin, carboxyhemoglobin, or sulfhemoglobin, should be considered as a potential cause of misleading oxygen saturation readings.

Glucose-6-phosphate dehydrogenase (G6PD) deficiency is the most common inherited enzymatic disorder of erythrocytes, affecting approximately 400 million individuals worldwide [1-3]. The enzyme plays a key role in the pentose phosphate pathway by generating nicotinamide adenine dinucleotide phosphate (NADPH), which protects erythrocytes against oxidative damage. Reduced G6PD activity increases susceptibility to oxidative stress and may precipitate hemolysis.

Methemoglobinemia results from the oxidation of hemoglobin iron from the ferrous to the ferric state, producing methemoglobin that is unable to effectively bind and deliver oxygen. Although arterial oxygen tension may remain normal, tissue oxygen delivery can be impaired [4-6]. In addition, dyshemoglobinemias interfere with conventional pulse oximetry, as abnormal hemoglobin species alter light absorption patterns, often leading to falsely low oxygen saturation readings that typically plateau around 85% [6-8].

The coexistence of G6PD deficiency and methemoglobinemia poses a relevant clinical dilemma. Methylene blue, the standard treatment for symptomatic methemoglobinemia, requires NADPH-dependent reduction to restore hemoglobin function. In patients with G6PD deficiency, this pathway is impaired, potentially rendering treatment ineffective and increasing the risk of hemolysis [9,10].

Chronic methemoglobinemia may be associated with congenital enzymatic deficiencies, hemoglobin variants, or prolonged exposure to oxidant agents. In contrast to acute forms, chronic presentations are often subtle and underrecognized. In the present case, prior suspicion of chronic methemoglobinemia was based on episodes of unexplained low oxygen saturation associated with preserved functional status, although formal co-oximetry confirmation had not been performed.

A saturation gap, defined as a discrepancy between low pulse oximetry readings and preserved arterial oxygen tension, represents a key diagnostic feature in the evaluation of suspected dyshemoglobinemia. Recognition of this finding is essential to avoid the misinterpretation of oxygenation status and inappropriate clinical interventions. Despite its clinical relevance, perioperative reports describing extreme pulse oximetry desaturation due to a saturation gap in patients with G6PD deficiency remain scarce.

## Case presentation

We report a case of marked pulse oximetry desaturation during general anesthesia in a patient with G6PD deficiency, ultimately attributed to a saturation gap consistent with methemoglobinemia. 

A 20-year-old man with known G6PD deficiency and suspected chronic methemoglobinemia was scheduled for an elective electrophysiologic study to evaluate recurrent supraventricular tachycardia. His past medical history was otherwise unremarkable. Home medications included propafenone and oral ascorbic acid. The patient denied dyspnea, fatigue, or exercise intolerance. Preoperative laboratory tests, including hemoglobin levels and metabolic parameters, were within normal limits.

**Figure 1:**
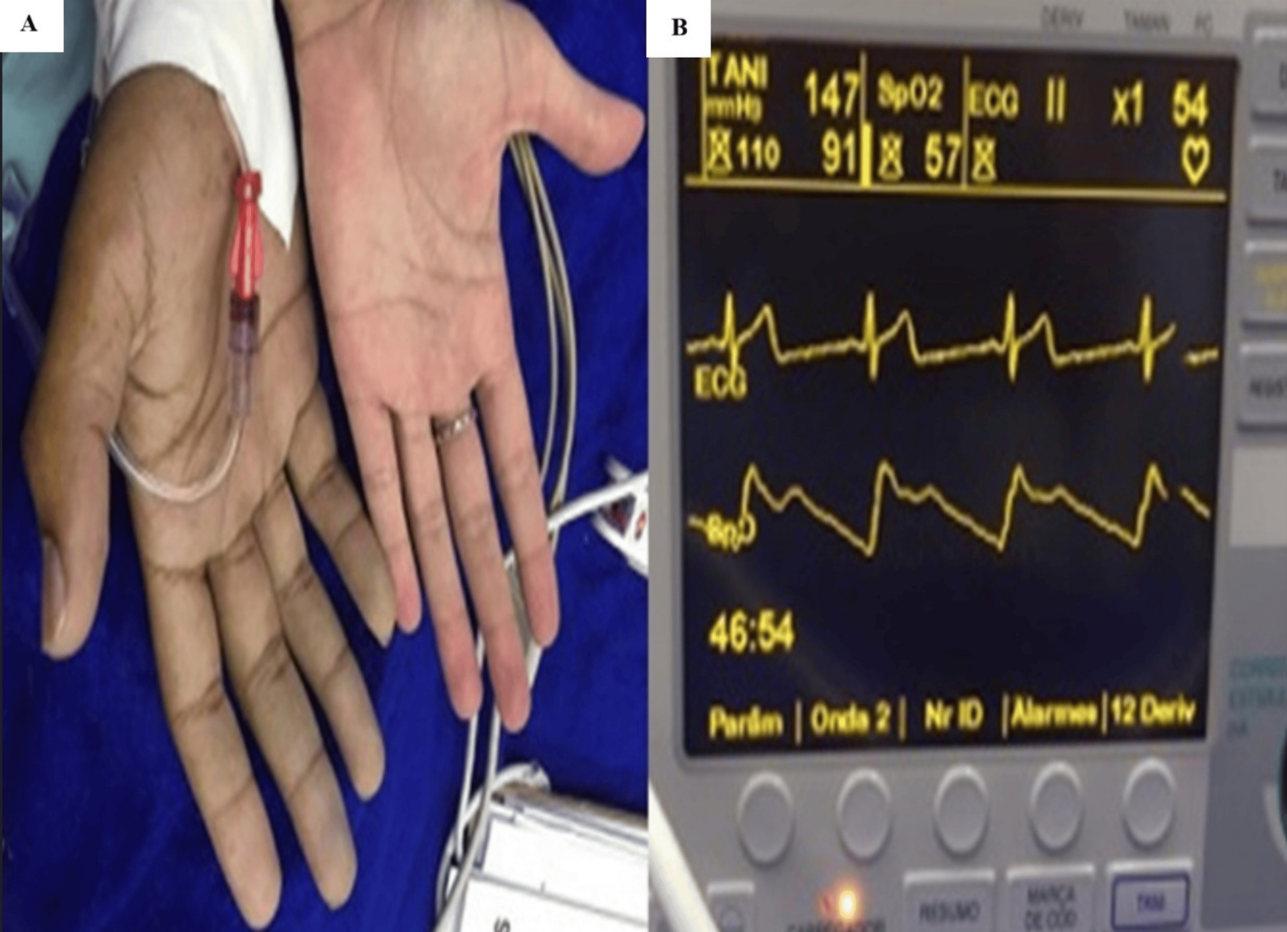
Clinical appearance and intraoperative monitoring findings in a patient with suspected methemoglobinemia (A) Grayish skin discoloration of the hands, consistent with dyshemoglobinemia. (B) Intraoperative monitor displaying markedly reduced pulse oximetry saturation (SpO₂) despite preserved hemodynamic parameters, illustrating the characteristic saturation gap.

Preoperative vital signs obtained prior to operating room transfer did not reveal significant abnormalities; however, pulse oximetry had not previously demonstrated values as low as those observed intraoperatively. The extremely low SpO₂ value (45%) is atypical for methemoglobinemia, in which saturation values usually plateau around 85%. This finding may reflect limitations of conventional pulse oximetry in the presence of dyshemoglobins, potential device-specific variability, or coexistence of additional optical interference factors (Figure 1). Despite this markedly low value, the patient appeared comfortable, alert, and hemodynamically stable, without signs of respiratory distress. Given the clear discrepancy between the pulse oximetry reading and the clinical presentation, dyshemoglobinemia was strongly suspected.

Arterial blood gas analysis obtained after the initiation of supplemental oxygen revealed a partial pressure of oxygen (PaO₂) greater than 400 mmHg, confirming preserved arterial oxygen tension despite the low pulse oximetry value. This discrepancy between pulse oximetry and arterial oxygenation characterized a saturation gap and was considered incompatible with true hypoxemia, guiding a conservative management strategy focused on close clinical monitoring rather than escalation of oxygen therapy. Based on prior evaluation and clinical findings, the methemoglobin fraction was suspected to be approximately 25%. Co-oximetry was not available at our institution. The combination of profound pulse oximetry desaturation, preserved arterial oxygen tension, characteristic discoloration, and previously documented methemoglobinemia supported the diagnosis.

Standard monitoring was applied, including continuous electrocardiography, non-invasive blood pressure monitoring, and pulse oximetry. In addition, bispectral index (BIS) monitoring was used to assess depth of anesthesia, and train-of-four (TOF) monitoring was employed to evaluate neuromuscular transmission. Urine output was also monitored throughout the procedure. An arterial catheter was inserted to allow continuous blood pressure monitoring and serial arterial blood gas analysis. General anesthesia was induced and maintained using target-controlled infusion of propofol (target concentration 3.5 µg·mL⁻¹) and remifentanil (0.2 µg·kg⁻¹·min⁻¹). A laryngeal mask airway was inserted, and mechanical ventilation was initiated with an inspired oxygen fraction of 0.6, maintaining stable ventilation parameters throughout the procedure.

During the two-hour electrophysiologic study, pulse oximetry values remained between 82% and 85%, consistent with the plateau typically observed in methemoglobinemia. Serial arterial blood gas analyses continued to demonstrate PaO₂ values exceeding 300 mmHg, with no evidence of metabolic acidosis or lactate elevation. Hemodynamic parameters remained stable throughout, and no arrhythmias or intraoperative complications occurred.

Given the known G6PD deficiency and the associated risk of hemolysis, methylene blue was not considered a safe therapeutic option. As part of contingency planning, the hyperbaric medicine team was alerted and remained on standby in the event of clinical deterioration or evidence of severe tissue hypoxia, as hyperbaric oxygen therapy would represent a potential alternative in this context.

The patient emerged from anesthesia uneventfully and was transferred to the recovery unit in stable condition. No clinical or laboratory evidence of hemolysis was observed in the postoperative period. Because the patient remained asymptomatic, with preserved oxygen delivery and stable hemodynamics, no specific treatment for methemoglobinemia was required.

At the outpatient follow-up three weeks after the procedure, the patient demonstrated complete resolution of the arrhythmia and remained clinically stable, with no evidence of complications related to redox imbalance or delayed hemolysis.

## Discussion

This case highlights the diagnostic significance of the saturation gap and underscores key considerations for anesthetic management in patients with G6PD deficiency.

The most notable feature was the presence of a saturation gap, defined by markedly reduced pulse oximetry values despite preserved arterial oxygen tension. When pulse oximetry readings are discordant with the clinical presentation, dyshemoglobinemia should be strongly suspected. Conventional two-wavelength pulse oximeters rely on standard hemoglobin absorption spectra; however, the presence of abnormal hemoglobin species, such as methemoglobin, disrupts these assumptions and may lead to misleading readings. Methemoglobin absorbs light at wavelengths similar to both oxyhemoglobin and deoxyhemoglobin, producing the characteristic plateau around 85% oxygen saturation, regardless of true arterial oxygen content [4-8].

In the present case, the markedly low SpO₂ value (45%) deviates from this expected pattern. Possible explanations include device calibration limitations, interference from dyshemoglobin fractions at higher concentrations, or additional optical artifacts. Importantly, this finding reinforces that pulse oximetry in dyshemoglobinemia should be interpreted with caution and always correlated with arterial blood gas analysis and clinical findings [4-8].

Several conditions may cause profound pulse oximetry desaturation in the presence of preserved arterial oxygen tension. Dyshemoglobinemias, including methemoglobinemia, carboxyhemoglobinemia, and sulfhemoglobinemia, should be considered in the differential diagnosis. In addition, technical factors such as poor peripheral perfusion, motion artifacts, nail polish, and ambient light interference may also produce falsely low readings. In contrast, true physiologic hypoxemia typically results in concordant reductions in both pulse oximetry saturation and arterial oxygen tension. Recognizing this distinction is essential when evaluating unexpected desaturation events [4-7].

The management of methemoglobinemia in patients with G6PD deficiency warrants particular caution. Methylene blue, the standard treatment for symptomatic methemoglobinemia, requires NADPH-dependent reduction to leukomethylene blue to restore hemoglobin function. In G6PD-deficient erythrocytes, NADPH production is impaired, which may render methylene blue ineffective and, importantly, increase the risk of hemolysis [1-3,9,10]. In the present case, methylene blue was withheld due to the patient's clinical stability and preserved oxygen delivery, as well as the potential risk of harm.

When methylene blue is contraindicated, alternative treatments include high-dose ascorbic acid and hyperbaric oxygen therapy, particularly in severe or symptomatic cases. Perioperative experience in patients with G6PD deficiency remains limited; however, available reports provide practical insights for clinical management. Across previously published cases (Table 1), both propofol-based total intravenous anesthesia and sevoflurane-based inhalational techniques have been used successfully. Favorable outcomes have been consistently reported when oxidant drugs are avoided and careful physiologic monitoring is maintained [11-18]. Adjunctive agents such as dexmedetomidine and short-acting opioids have also been used to enhance hemodynamic stability and reduce anesthetic requirements [13-16].

**Table 1:** Reported perioperative cases of anesthesia in patients with G6PD deficiency G6PD: glucose-6-phosphate dehydrogenase; TIVA: total intravenous anesthesia

Author (year)	Procedure	Anesthetic technique	Key perioperative considerations	Outcome
Abreu et al. (2002) [11]	General surgery	TIVA + spinal anesthesia: midazolam + propofol + fentanyl + bupivacaine	Avoidance of oxidant drugs	Uneventful
Cho et al. (2017) [12]	Robot-assisted laparoscopic surgery	General anesthesia (sevoflurane + fentanyl + rocuronium)	Careful monitoring (thermal and CO₂ control); avoidance of oxidative stress	Uneventful
Takahashi et al. (2017) [13]	Pediatric frenectomy	Dexmedetomidine + propofol infusion	Sedation without oxidant medications	Uneventful
Sharma et al. (2018) [14]	Infant surgery	Propofol infusion	Careful hemodynamic monitoring	Uneventful
Wang et al. (2018) [15]	Surgical procedure in a patient with favism	TIVA (propofol + remifentanil + dexmedetomidine)	Avoidance of triggering medications	Uneventful
Goi et al. (2019) [16]	Dental surgery	Propofol + remifentanil + mepivacaine	Standard monitoring; avoidance of oxidative stress	Uneventful
Cicvarić et al. (2022) [17]	Pediatric surgery	Sevoflurane + sufentanil	Perioperative monitoring and drug selection	Uneventful
Haka et al. (2024) [18]	Adenoidectomy/tonsillectomy	Midazolam/sevoflurane + propofol	Avoidance of oxidant agents	Uneventful

Although current evidence remains limited, it suggests that commonly used anesthetic agents can be safely administered in patients with G6PD deficiency, provided that oxidative stressors are minimized. This supports a pragmatic perioperative approach focused on avoiding known triggers of hemolysis while ensuring adequate oxygen delivery and hemodynamic stability [11-18]. Relevant oxidant drugs in the perioperative setting include local anesthetics such as benzocaine and prilocaine, nitrates, dapsone, sulfonamides, and certain antibiotics. Avoidance of these agents is essential in patients with G6PD deficiency or suspected methemoglobinemia.

These findings further emphasize the diagnostic significance of the saturation gap and underscore key considerations for anesthetic management in patients with G6PD deficiency. Maintaining adequate oxygenation in patients with dyshemoglobinemia remains a recognized perioperative challenge, particularly when conventional monitoring tools are unreliable [19]. Importantly, this finding reinforces that pulse oximetry in dyshemoglobinemia should be interpreted with caution and always correlated with arterial blood gas analysis and clinical findings [4-8]. Recent evidence further highlights that low pulse oximetry readings do not necessarily reflect true hypoxemia in the presence of dyshemoglobinemia, reinforcing the need for careful clinical interpretation [20]. Within this context, the unique contribution of this case lies in the description of extreme pulse oximetry desaturation (SpO₂ 45%) associated with a saturation gap in a clinically stable patient, highlighting the limitations of conventional monitoring and reinforcing the importance of clinical reasoning in perioperative decision-making. Accordingly, recognition of the saturation gap, together with preserved arterial oxygenation and clinical stability, enabled a conservative and safe perioperative approach, preventing unnecessary and potentially harmful interventions.

An important limitation of this report is the absence of co-oximetry, which precluded the direct quantification of methemoglobin levels and required reliance on indirect clinical and laboratory findings. This also reflects a broader limitation in perioperative care, as pulse oximetry alone may be unreliable in the presence of dyshemoglobinemia and can lead to the misinterpretation of oxygenation status and inappropriate interventions. Co-oximetry, which directly measures dyshemoglobin fractions, remains the gold standard for diagnosis [6-8]. In settings where it is unavailable, arterial blood gas analysis combined with careful clinical assessment is essential for accurate interpretation. Additionally, the lack of serial measurements limits a more detailed analysis of intraoperative dynamics.

## Conclusions

This case highlights the clinical importance of recognizing a saturation gap during anesthesia, particularly in patients with G6PD deficiency and suspected dyshemoglobinemia, where marked pulse oximetry desaturation may occur despite preserved arterial oxygenation. Such findings should prompt the consideration of abnormal hemoglobin species to avoid misdiagnosis and unnecessary escalation of care. Perioperative management requires a cautious and individualized approach, as standard treatments such as methylene blue may be contraindicated due to the risk of hemolysis; in this context, conservative management with close monitoring and maintenance of adequate oxygen delivery proved safe and effective, with hyperbaric oxygen therapy available as a contingency. The favorable perioperative course, absence of hemolytic complications, and successful use of propofol-based anesthesia reinforce that careful clinical assessment, avoidance of oxidative stressors, and appropriate diagnostic integration are essential for safe management in complex cases involving dyshemoglobinemia.
